# SOCIAL DETERMINANTS OF HEALTH AMONG CUSTODIAL GRANDMOTHERS: ADVANCING AN INTERSECTIONAL APPROACH

**DOI:** 10.1093/geroni/igae098.3219

**Published:** 2024-12-31

**Authors:** Tenesha Littleton

**Affiliations:** University of Alabama, Tuscaloosa, Alabama, United States

## Abstract

Racial disparities in custodial grandparenting, as well as disparities in the physical and mental health outcomes of custodial grandparents, are well-documented. As a result of long-standing socio-structural inequalities across the life course, custodial grandparents are disproportionately single, Black women with low socio-economic status. These categories of difference and the process of racialization contribute both to the need for grandparent caregiving in the Black community and the negative health consequences of caregiving for Black custodial grandmothers. Macro level structures and systems of oppression impact access to the social determinants of health shaping the life trajectories of racialized groups and contributing to unequal aging which suggests the importance of an intersectional lens when understanding the lived experiences of Black custodial grandmothers. A scoping review was conducted to determine the extent to which an intersectional framework is utilized in research examining the experiences of Black custodial grandmothers. A search of relevant databases (PsychINFO, SociINDEX, PubMed, and Web of Science) was performed using the following search terms and Boolean logic: (“grandparent” OR “grandmother” OR “grandfather” OR “custodial grandparent”) AND (“intersectionality”). Quantitative, qualitative, and mixed-methods peer-reviewed journal articles published in English through January 2024 were included. Findings suggest that research integrating intersectionality with large representative samples is limited. This presentation will propose a conceptual framework that integrates the social determinants of health with an intersectional life course perspective as proposed by Ferrer and colleagues (2017). Recommendations for future research using intersectional approaches will be offered.

